# The Association between Assisted Reproduction Technology (ART) and Social Perception of Childbearing Deadline Ages: A Cross-Country Examination of Selected EU Countries

**DOI:** 10.3390/ijerph18042111

**Published:** 2021-02-22

**Authors:** Eun Jung Kim, Min Jung Cho

**Affiliations:** 1 School of Architecture, Hanyang University, Seoul 04763, Korea; uwcsea0620@hotmail.com; 2 Faculty Governance and Global Affairs, Leiden University College, 2595 DG The Hague, The Netherlands

**Keywords:** social perception of childbearing deadline ages, advancement of assisted reproductive technology, maternal and paternal childbearing ages

## Abstract

The advancement of assisted reproductive technologies (ART) has gained much attention in relation to childbearing postponement. Our study’s purpose was to empirically examine how perceptions of childbearing deadline age vary in association with availability and prevalence of ART across different countries. The present study used data from the 2006 European Social Survey and the 2006 European Society of Human Reproduction and Embryology to examine selected EU countries. A total sample of 17,487 respondents was examined. Multilevel regression modeling was used. Results showed that first, younger generations were more generous with maternal childbearing ages but stricter with paternal deadline ages. Second, respondents residing in countries with higher percentage of reproductive clinics per population were more generous with maternal ages, however no significant association was observed with regard to paternal childbearing ages. Third, on the contrary, respondents residing in countries with higher utilization of ART treatments were stricter with maternal ages, which may be because they are more likely to be aware of the physiological and financial difficulties associated with ART treatments. The present study is meaningful in that it is the first study to empirically examine social perceptions of childbearing ages in relation with ART.

## 1. Introduction

The entry into parenthood has been increasingly delayed in many developed countries [1,2,3,4,5]. Parenthood in industrialized societies is characterized by arguably irreversible delays in childbearing due to lifestyle changes such as increases in women’s participation in the labor market and increased years of education [6]. The nexus between social and biological fertility remains an important debate as social norms play a powerful role in shaping expectations for parenthood and what is perceived as acceptable in various stages throughout the life course [7,8,9]. For example, in most industrialized countries, childbearing during adolescence is considered socially deviant regardless of biological fertility. Perceptions of the suitable time for parenthood are part of a certain culture of normalcy in terms of what is expected for adults [10,11] and this culture influences fertility behaviors. It is expected that if a social deadline age for childbearing is lower than the upper limit of the biological age range (usually between 40–50), actual reproduction is likely to be influenced by the social age.

Social childbearing deadline age is a social construct, and thus is susceptible to influence from social changes [12,13,14,15,16]. In recent years, assisted reproductive technologies (ART) has gained much attention in relation to childbearing postponement. The provision and use of fertility treatments have likely affected perceptions of childbearing ages [17,18,19]. However, arguably, not many studies have looked into ART and its association with social perception of childbearing ages [19,20,21,22].

While existing research on ART has focused on costs and difficulties associated with ART treatments, ART-related births to fertility, and effectiveness of ART, only a few studies have focused on social perception of childbearing age [23,24,25,26,27,28]. However, these few studies are limited because they are based on single country and are qualitative literature reviews. Social norms and perceptions of childbearing age are important as they may set the limits on policies of healthcare provision and the societal climate regarding ART may vary between and within countries. Also, although men contribute to half of infertility causes, studies on ART have largely been focused on female infertility [26]. This gender imbalance and bias in reproductive health and reproductive health studies are likely to negatively reinforce people’s perception of infertility as women’s responsibilities. Hence, it is important to examine the association of ART and childbearing deadline ages of both genders.

Using the 2006 European Social Survey, the present study examined European countries and explored how ART influences perceptions of both maternal and paternal childbearing deadline ages quantitively. Although the data comes from 2006, we believe the results are meaningful in that it provides vital insight and foundation into understanding people’s perceptions of childbearing deadline ages and how the advancement of ART influence people’s perceptions. Also, by examining both maternal and paternal childbearing deadline ages, we hope our findings help facilitate women’s empowerment and gender equality in reproductive health and fertility treatments. We hypothesize that individuals residing in countries with higher availability and prevalence of ART are more likely to be generous with childbearing deadline ages than individuals residing in countries with relatively lower availability and prevalence of ART.

## 2. Data and Methods

### 2.1. Data

#### 2.1.1. Individual-Level Data

Data at the individual level were all drawn from the 2006 European Social Survey (ESS) [29]. The ESS surveyed populations aged 15 and over in 25 European Union countries: Austria, Belgium, Bulgaria, Cyprus, Denmark, Estonia, Finland, France, Germany, Hungary, Ireland, Latvia, Netherlands, Norway, Poland, Portugal, Romania, Russia, Slovakia, Slovenia, Spain, Sweden, Switzerland, Ukraine and the UK.

The ESS is a bi-annual survey conducted using face-to-face interviews. To enhance comparability, the same sampling plan was applied in each country and the questionnaires were carefully translated with sensitivity to country contexts. In each country, the national funding agency appointed a National Coordinator (NC) and a survey organization to implement the survey according to the common ESS Specification, which is set to ensure accuracy of data in each country and to optimize comparability of data across countries. The survey was conducted for at least 6 weeks between September 2006 and January 2007, depending on the schedule of the surveyed country. A strict random probability sampling method was applied to ensure representation of each national population. Response rates varied between 46% (France) and 73% (Portugal and Slovakia) [13]. Final sample sizes varied from as low as 995 individuals (Cyprus) to a maximum of 2916 individuals (Germany). Post-stratification weights were provided to adjust for sampling error and non-response bias, which were applied throughout the analysis in this study [29]. 

The ESS applied a split ballot design, where the sample was randomly split and approximately half of the respondents were asked questions about women and the other half were asked the same questions, but about men (i.e., the word ‘women’ was substituted for the word ‘men’). Hence, a total of 17,487 respondents were interviewed about women’s childbearing ages and a total of 16,992 respondents about men’s childbearing ages. In addition to data for individuals’ perception of childbearing deadline ages, all other individual-level control variables from the ESS were also employed.

#### 2.1.2. Country-Level Data

Four different data sources were used for country-level factors. First, to examine countries’ availability and prevalence of ART, we used the 2006 European Society of Human Reproduction and Embryology (ESHRE) annual report [30]. The ESHRE covers a range of ART measurements. In the present studies, we used the number of IVF clinics, the number of ART cycles, and the number of ART infants per total births in a country. Data for some countries were missing in the ESHRE report and hence in this study, we were only able to employ data for 21 countries, 13 countries, and 12 countries for number IVF clinics, ART cycles, and ART infants, respectively. In this study, only countries with available data were used in the final analyses (see Appendix A for the list of countries by each variable).

To control for country-level confounding factors, the present study controlled for countries’ GDP per capita, female labor force participation rate, and the family and child government benefit spending per GDP. To correspond with the individual-level ESS data, all the country-level data were collected from year 2006. Data for GDP per capita [31] and female labor force participation rate were obtained from the 2006 World Bank data [32] and family and child government benefit spending were obtained from the 2006 Eurostat Social Protection Statistics [33].

### 2.2. Measures

#### 2.2.1. Dependent Variables

The goal of this paper was to examine how a country’s availability and prevalence of ART impact individuals’ perception of childbearing deadline ages. In this paper, we examined both maternal and paternal childbearing deadline ages. The ESS asked respondents “After what age would you say a woman [or man] is generally too old to consider having any more children?” Interviewers were instructed to explain that ‘having any more children’ referred to either the first or any additional children a person may have. Childbearing deadline ages were measured as continuous variables.

#### 2.2.2. Independent Variables

Three independent variables were used to measure the availability and prevalence of ART in a country: (1) number of IVF clinics, (2) number of ART cycles, and (3) percentage of ART infants per national births. First, the number of IVF clinics was measured as population (thousand) per IVF clinics, which indicates the availability of IVF clinics in relation to the population size of a country. Higher population per IVF clinic indicates that there are fewer IVF clinics available in a country. Second, ART cycles were measured as the number of people who have utilized ART per population (million). The variable represents the prevalence of usage of ART in relation to the population size of a country. Higher values indicate that more people have used ART in a country. Lastly, we used the percentage of ART infants per national births to measure the occurrence of ART infants in the country.

#### 2.2.3. Control Variables

The present study controlled for seven individual-level demographic characteristics and three country-level factors. The individual-level control variables were age group, sex, marital status, educational attainment, residence location, religion, perceived health, perceived subjective household income, and number of children. The ESS questionnaire asked interviewers to code the “sex” of the respondents as either “female” or “male” and the present study decided to follow the exact phrasing of the ESS questionnaire, instead of “gender”. Also, the ESS measured educational attainment based on the International Standard Classification Education (ISCED), an international framework designed by the United Nations to facilitate comparisons of education systems across countries. The ISCED is divided into six levels (0 = pre-primary education, 1 = primary education, 2 = lower secondary education (or middle school), 3 = upper secondary education (or high school), 4 = post-secondary non-tertiary education, 5 = first stage tertiary education (i.e. Bachelor and Master degree equivalent), 6 = second stage tertiary education (i.e. Ph.D. degree equivalent) [34]. Here, we focused on four broad categories of educational attainment: “Less than lower secondary (ISCED 0–1)”, “Lower secondary completed (ISCED 2)”, “Upper secondary completed (ISECD 3)”, and “Higher than post-secondary completed (ISECD 4–6).” All individual-level characteristic control variables were measured as categorical variables. The three country-level variables were GDP per capita (measured as 2010 constant US $1000), female labor force participation rate (aged 15–64), and family and child benefit spending per GDP (measured as a percentage). All country-level variables were measured as continuous variables and based on the year 2006 to correspond with the ESS data. The 2006 GDP per capita was reported in 2010 USD because the World Bank only provides the data either in 2010 or the latest current (as of now, 2021) USD. In the present study, we decided to use GDP per capita in constant 2010 USD because the latest current data is likely to change soon and may be confusing to readers when they read the article since the data is to be updated every year. (Please see Table 1 for detailed calculation).

### 2.3. Modeling

Multilevel regression modeling was used to examine the association between a country’s ART availability and prevalence and social childbearing deadline ages. Since our ESS data are from individuals nested within countries, if we were to run a single-level simple OLS regression model, the standard errors would be underestimated, leading to an overstatement of statistical significances [35].

Six separate multilevel regressions were conducted. The first and second regressions examined the relationship between the availability of IVF clinics and perception of childbearing deadline age for mothers and fathers, respectively. The third and fourth regressions examined the relationship between ART cycle and perception of childbearing deadline age for mothers and fathers. Lastly, the fifth and sixth regressions examined the relationship between the percentage of ART infants and perception of childbearing deadline age for mothers and fathers. The final model for the present study was as follows:

Y_ij_ = β_0_ + β_1_ART_j_ + β_2_C_j_ + β_4_X_ij_ + u_0j_ + ε_ij_ (1)

Here, individual perception of childbearing age was denoted by Y_ij_, where i represents a given individual and j represents a given country. Individual-level control variables were denoted as X_ij_ and country-level control variables were denoted as C_j_. The independent variable was represented as ART_j_. Lastly, ε_ij_ represents random error independent of the random parameter, u_0j_.

## 3. Results

### 3.1. Descriptive Results

Perceptions of maternal and paternal childbearing deadline ages varied by country. Perception of maternal deadline ages ranged 39–44, and paternal deadline ages ranged 45–51, respectively. Austria was most generous with both maternal and paternal deadline ages. Hungary was the strictest with maternal childbearing age and Denmark was the strictest with paternal childbearing deadline age. Overall, on average, people perceived the childbearing deadline age for women as 42 years old, and for men, 48 years old.

Descriptive results showed that there are significant differences in perceptions of maternal and paternal childbearing deadline ages by age groups. Results showed that younger people tend to be more generous with maternal childbearing ages and stricter with paternal childbearing ages than older people. Male respondents were significantly more generous with maternal childbearing deadline ages than female respondents, while female respondents were significantly more generous with paternal childbearing deadline ages than male respondents. Respondents with higher educational attainment were significantly more generous with both maternal and paternal childbearing deadline ages than respondents with lower educational attainments. For further details on how people’s perceptions of maternal and paternal childbearing deadline ages differ by demographic characteristics, see Table 2. 

With regard to availability and prevalence of ART, data showed that IVF clinics were most available in Switzerland and least available in Ukraine. Regarding ART cycles, Denmark had the highest usage of ART per capita and Austria had the lowest. Lastly, on average, the percentage of infants born by ART was highest in Denmark and lowest in Austria. See Table 3 for further details.

### 3.2. Bivariate Results

**Figure 1:**
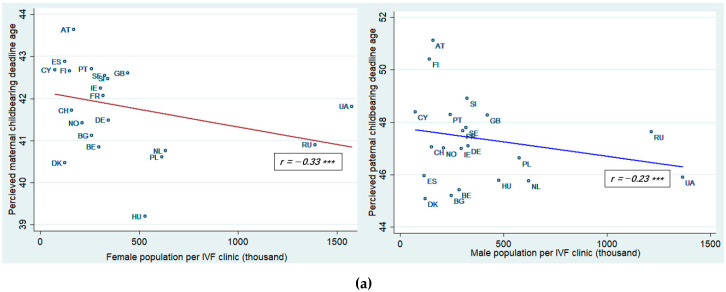

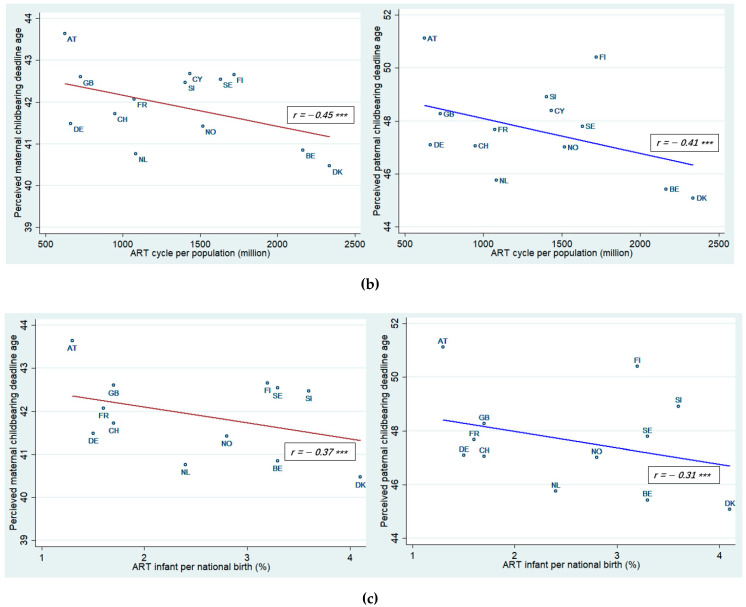
(**a**) Association between social perception of maternal and paternal childbearing deadline ages and availability of IVF clinics; (**b**) Association between social perception of maternal and paternal childbearing deadline ages and ART cycle; (**c**) Association between social perception of maternal and paternal childbearing deadline ages and ART. Pearson correlation (*r*) was conducted to examine the bivariate relationship (*** *p* < 0.001). See Appendix B for the list of country abbreviations.

The bivariate relationship between countries’ population per IVF clinics and maternal childbearing (*r* = −0.33, *p* < 0.001) and paternal childbearing (*r* = −0.23, *p* < 0.001) ages showed both showed significant negative relationships, which indicate that people perceived significantly higher maternal and paternal deadline ages when there were more IVF clinics available in a country (see Figure 1).

Second, the results showed significantly negative relationships between ART cycles and social childbearing deadline ages (maternal: *r* = −0.45, *p* < 0.001; paternal: *r* = −0.41, *p* < 0.001). These results contradict our hypotheses and indicate that individuals who reside in countries with higher ART usage were more likely to perceive lower maternal and paternal childbearing deadline ages.

Lastly, the results also showed significant negative relationships between the percentage of ART infants and social childbearing deadline ages (maternal: *r* = −0.37, *p* < 0.001; paternal: *r* = −0.31, *p* < 0.001). Individuals who reside in countries with higher ART infant percentages were more likely to perceive lower maternal and paternal childbearing deadline ages, which also contradicts our initial hypotheses, that countries with higher numbers of infants born by ART would have higher perceived maternal and paternal deadline ages.

### 3.3. Multivariate Results

The multivariate results showed that there was a significant negative association between IVF clinics and maternal childbearing deadline ages even after controlling for other individual and country level covariates (b = −3.16 × 10^−3^, *p* < 0.05); that is, individuals residing in countries with higher availability of IVF clinics were significantly more likely to be generous with maternal childbearing deadline ages even after controlling for other demographic and country-level factors. On the other hand, the paternal deadline age was no longer significantly related with IVF clinics after controlling for other factors (see Table 4). Second, results indicated a significant negative association between ART cycles and maternal childbearing deadline age (b = −1.02 × 10^−3^, *p* < 0.05). That is, individuals perceived significantly lower maternal childbearing deadline ages in countries with higher usage of ART. Similarly, paternal childbearing deadline age was shown not to be significantly associated after controlling for other factors. Lastly, the association between percentage of ART infants per national births and individuals’ perception of childbearing deadline ages became statistically insignificant after controlling for other covariates for both maternal and paternal ages.

## 4. Discussion

This study examined the association between availability and prevalence of ART and social perception of childbearing deadline ages. Here, we examined ART by: (1) number of IVF clinics, (2) number of ART cycles, and (3) percentage of ART infants per national births of a country. Our findings suggest that individuals residing in countries with higher availability of IVF clinics were significantly more likely to be generous with maternal childbearing deadline ages even after controlling for other covariates. However, individuals residing in countries with higher ART utilization rates were significantly likely to be stricter with maternal childbearing deadline ages. Paternal childbearing deadline age was not statistically associated with either variable.

### 4.1. Limitations

First, the results and interpretation of our study are limited to selected EU countries that have reported data on IVF clinics, ART cycles, and ART infant births. Therefore, the results may be different if we were to conduct the study, for example, in other countries or continents such as Asia. Moreover, our study is a single-year cross-sectional study based on 2006 data. Hence, people’s perceptions of social childbearing ages may be different if the survey was conducted in 2021. Caution must be used in interpretation of cause and effect due to lack of investigation into temporal trends. To this date, no longitudinal dataset exists for Europe or elsewhere that would allow us to compare changes in perceptions of age norms over time.

Second, since the survey was conducted, there has been a significant advancement in the field of ART, including the introduction of alternative and novel approaches such as the intra-ovarian autologous platelet-rich plasma (PRP) infusion [36] and the improvement of gamete/embryo culture techniques [37]. However, despite the advancement of such technology, reservations exist among clinical practitioners as evidence so far is based on case study reports and uncontrolled before-after studies with limited randomized control trial results or moderate to low quality of evidence [36,37]. While the advancement of technology is promising to those who are trying to conceive, a cautionary approach must be taken due to the controversy surrounding add-ons in assisted reproduction [38]. To what extent these technologies have impacted the perception of social aging is yet to be known and future research is warranted to examine both the exposure and take-up of these novel approaches in medicine in our society.

Third, cross-border reproductive care has also been increasing since the survey was conducted in 2006 [39]. However, due to data limitations, we were unable to cover this area in our study. Studies show that there could be approximately 24,000−30,000 cycles of cross-border treatments, involving 11,000–14,000 patients in Europe annually [40]. Possible reasons for cross-border reproductive include restrictions on access to certain forms of treatment in the home countries; high cost of treatments in the home countries; a desire for donor anonymity; sex or trait selection; a lack of expertise in the home countries’ long waiting time, etc. [40]. For future research, the authors call for studies on cross-border reproductive care, fertility, and social perceptions. Yet, for this to be possible, it is important to first develop a cross-border co-management of national data exchange platform.

Lastly, the COVID-19 pandemic is expected to have a substantial effect on fertility treatment and fertility. Affordability and availability of treatment are two of the most important factors in couples’ decisions to pursue ART [41]. The emergency of COVID-19 has restricted access to IVF clinics. On March 2020, the American Society for Reproductive Medicine recommended suspension of initiation of IVF treatments and at least 85% of clinics have shut down provision of routine care in the US as recommended [41]. Also, economic impact due to COVID-19 is expected to have a significant impact on the downfall of IVF treatments based on the evidence from the 2008 financial crisis, which caused a four-year plateau in infertility treatments with predicted 53,000 fewer IVF cycles and 17,000 fewer births [41]. Updated researches should take into consideration of the COVID-19 pandemic and its impact on IVF treatments.

Despite these limitations, a major strength of our study is that the database of European Social Survey database comprises a large and representative sample of various European countries, as well as individual socio-demographic characteristics. It also is the first study to empirically examine ART and social perceptions of childbearing age deadlines. Also, findings from our study may offer insight into understanding perceptions of childbearing ages in 2021 and future. For example, according to the European IVF Monitoring (EIM) consortium, the number of countries and clinics providing data to the EIM increased from 482 clinics (1997) to 1064 (2011) [42]. These clinics reported that the total number of cycles increased from 203,225 (1997) to 609,973 (2011) which indicates increased use of ART across Europe [42]. Furthermore, findings from our study can provide a foundation for future studies in ART and fertility age. For instance, a recent 2019 study by Fauser et al. [43] examined the public perception of IVF treatment in six Western European countries and found that that 54% of the respondents have considered or would consider having IVF treatments and 52% answered that the availability of IVF treatment would encourage them to delay conception. Fauser et al. [43] reported that this delay in conception response was especially pronounced in France and Germany, but the authors did not provide a concrete analysis of this outcome. The study was focused on descriptive findings and did not look at the relationship between the two variables. Although changes since 2006 would have to be taken into account, our study provides insights into understanding why respondents in France and Germany may have been more generous in later childbearing in Fauser et al.’s study [43], possibly in response to the high availability and utilization of ART services in those countries based on our study. In addition, because our study examined ART in several dimensions, including the availability of ART clinics, ART usage, and ART births, it provides a relatively comprehensive overview that can support future studies of ART, age norms, and how the advancement of ART may influence fertility behaviors.

### 4.2. Implications

Our study results support our hypothesis that perceived maternal childbearing ages have increased as technologies to assist infertility have developed and become more accessible, i.e., number of IVF clinics. However, no significant association was observed with regards to an individuals’ perception of paternal childbearing deadline age and number of IVF clinics. A possible explanation for such an outcome may be due to lack of understanding of male infertility and use of ART treatments. Although studies show that men contribute to more than half of infertility causes [44], people generally still think that childlessness is primarily a women’s issue [25], and even though ART is used to tackle both male and female infertility, it is often perceived to be used primarily for female reproductive needs [7,27,45]. While late maternal age has been a longstanding topic of research, late paternal age has been relatively studied less [46]. Of the few studies, results showed that late fatherhood also have a significant negative effect on hormone levels, fertility, and sperm quality [47,48], and significantly decrease IVF success rates. de La Rochebrochard et al. [49] examined male patients treated by IVF in France, 2005 and discovered that the treatment success rates of male patients decreased significantly after aged 40. Also, Throsby and Gill in 2004 interviewed UK husbands, who were diagnosed with infertility but discontinued using IVF treatments, and found that husbands stop IVF treatments because they felt that infertility and IVF treatments threatened their masculinity and their wives were being pitied [50]. As a result, the authors presume that social gender bias exists in the perception of the use of IVF clinics and ART in general.

Contrary to the authors’ expectation, results indicated that individuals residing in countries with higher utilization of ART treatments (i.e., ART cycles) were likely to be stricter with maternal childbearing deadline ages than individuals residing in countries with lower ART use. Two explanations are possible. First, the small sample size may have contributed to such findings: whilst 21 countries were examined for IVF clinics, only 13 countries were examined for ART cycles. Hence, the results are more likely to be influenced by outliers. For example, despite a relatively high usage of ART cycles in Denmark, individuals residing there reported relatively low maternal childbearing deadline ages on average. Another potential explanation is that individuals from countries with high ART utilization rates are more likely to be aware of the physical and psychological burden of ART treatments and this may negatively influence perceptions of late pregnancy and postponement. ART treatments can often be physically, emotionally, and economically draining [51]. Studies show that ART treatment cycles induce significant stress, especially for women [52]. Gamerio et al.’s study reported the psychological burden and relational and personal problems associated with ART treatments [53]. As ART is emotionally and physically demanding, individuals and couples going through infertility cope with the challenges through social support (i.e., friends and families). Peterson et al. [54] surveyed men and women, diagnosed with infertility and referred to university-affiliated hospitals for IVF from 1995–2001, to examine infertility stress and found that whilst women sought more to social support (i.e., talking to friends and families about the infertility) to cope with infertility stress compared with men, men sought more to self-controlling (i.e., keeping feeling about the infertility to oneself and trying to keep these feelings from interfering with daily activities) and distancing (i.e., making light of the infertility). Based on such results, the authors assume that individuals residing in countries with higher ART usage may be more aware of the emotional and physical burdens of ART treatments, especially for women, as described to them by friends or family members who have experienced ART treatments. Hence, social sentiments and perceptions against late maternal childbearing may also be more negative. On the other hand, with regard to ART cycles and paternal childbearing deadline ages, our multivariate results showed that there was no statistically significant association, which may be because men are less likely to talk about their fertility issues and treatment experiences than women. Further studies are needed to confirm this hypothesis.

Results showed that the percentage of ART infants per national births was not statistically significant in individuals’ perception of either maternal or paternal childbearing deadline ages after controlling for other factors. This may be because the rate of ART infants per national births is relatively low (i.e., 1.3–4.1%), perhaps too low to significantly affect public perceptions. The association between ART infants and individuals’ perception of childbearing deadline ages disappeared once other factors were considered. Our multivariate analyses showed that individual demographic factors such as age, marital status, education, health, and number of children had a more significant effect in determining people’s perception of childbearing deadline ages than a country’s number of ART infants.

Lastly, interestingly, our multivariate results indicated an especially strong association between respondents’ ages and their perceptions of childbearing deadline ages. Results showed that younger respondents were significantly more generous with maternal childbearing deadline age but stricter with paternal childbearing deadline age. These results indicate differences in the perception of childbearing and gender roles across generations. First, younger generations are more likely to be understanding of late marriage and more knowledgeable about ART than older generations and hence, they are more likely to be generous about late motherhood than older generations. In the EU, for instance, the share of births to mothers aged 40 or over has increased from 1.6% in the late 1980s to 3.0% in 2006 [55]. Also, some studies denote that older mothers tend to be in more stable relationships, are more highly educated, and are more economically sound than younger mothers, and hence, tend to provide a more stable and positive environment for children than younger mothers [56,57]. Second, on the contrary, younger generations were stricter with late fatherhood than older generations. The authors assume that this is because younger generations perceive fatherhood more in relation to childrearing capability than biological capability. In fact, according to Finley’s study adolescents born to fathers who were aged 40 or over evaluated their fathers’ parental quality lower than those whose fathers were aged 30–39 [58]. Whilst older generations considered paternal childbearing age primarily based on biological capabilities, younger generations view childbearing age in relation to whether one can adequately raise a child [59,60,61].

## 5. Conclusions

The present study is the first to empirically examine the association between the availability and prevalence of countries’ ART and people’s perception of childbearing deadline ages. The authors understand that the data is relatively old, however, until now, it is the only quantitative data that examines people’s changing perception of childbearing ages, especially across multiple countries. However, findings from the study provide important insights into understanding people’s perception of childbearing deadline ages and reproductive technology. First, in this study, we discovered that younger generations were more generous with maternal childbearing ages but stricter with paternal childbearing ages. Second, we discovered that respondents residing in countries with higher number of IVF clinics were significantly more generous maternal childbearing ages but the association was not significant with regard to paternal childbearing ages, which indicate that although ART is used to tackle both male and female infertility, social perceptions think it is primarily for female infertility. Third, we discovered, contrary to our expectations, respondents residing in countries with higher utilization of ART treatments were stricter with late maternal childbearing ages, which, we assume maybe because they are more likely to be aware of the physiological and financial difficulties of ART treatments. Further studies are needed to explore this matter. As the first study to explore social perceptions of childbearing deadline age in relation to ART availability and prevalence, this study has laid the foundation for further research in this area. Overall, the present study discovered that gender bias exists in the perception of the use of IVF clinics and ART. As only a limited number of studies have explored ART and gender norms [62,63], it will be interesting to see if this gender bias persists in the future as male use of ART increases and efforts to improve gender equality continue. Although in our study, the advancement of ART and increased IVF clinics have shown to relax public perception of late motherhood, this result may be a reflection of the gender bias in people’s perception that infertility treamtments are primarily for women and the advancement of ART may possibly also act as a reinforcer to gender bias. We hope our study raise awareness on this issue and contribute to enhancing women’s autonomy and empowerment in reproductive health. We call for future updated studies to explore that cover a more diverse set of countries and examine how the relationships change over time.

## Figures and Tables

**Table 2:** Respondents’ perception of maternal and paternal childbearing deadline ages by demographic characteristics.

Respondents’ Demographic Characteristics	Mean (SE)	
	Maternal Childbearing Deadline Age	Paternal Childbearing Deadline Age
Age-group		
15–24	42.3 (0.16)	46.6 (0.18)
25–34	42.2 (0.13)	47.1 (0.17)
35–44	42.3 (0.10)	47.8 (0.19)
45–54	41.7 (0.10)	47.4 (0.17)
55–64	41.2 (0.10)	47.7 (0.18)
65 +	41.2 (0.09)	47.4 (0.15)
Gender		
Female	41.7 (0.06)	47.5 (0.09)
Male	41.9 (0.07)	47.1 (0.11)
Health		
Good or Very Good	42.1 (0.06)	47.5 (0.08)
Fair	41.3 (0.08)	47.1 (0.15)
Bad or Very Bad	41.2 (0.15)	46.7 (0.25)
Marital Status		
Married or Civil partnership	41.6 (0.06)	47.2 (0.09)
Single and not in civil partnership (including divorced, separated, widowed)	42.1 (0.07)	47.5 (0.10)
Educational Attainment Completed		
Less than lower secondary (ISCED 0–1)	41.7 (0.12)	47.1 (0.21)
Lower secondary completed (ISCED 2)	41.7 (0.11)	46.5 (0.16)
Upper secondary completed (ISCED 3)	41.6 (0.07)	47.3 (0.11)
Higher than post-secondary completed (ISCED 4–6)	42.2 (0.08)	48.1 (0.13)
Number of Children		
None	42.4 (0.09)	47.5 (0.12)
1	41.7 (0.10)	47.2 (0.16)
2	41.4 (0.07)	47.0 (0.12)
3 or more	41.6 (0.09)	47.5 (0.16)
Residence		
City	41.9 (0.06)	47.4 (0.09)
Rural or country	41.8 (0.07)	47.2 (0.11)
Religion		
Do not belong to any religion	41.9 (0.07)	47.0 (0.11)
Roman Catholic	41.9 (0.08)	47.5 (0.12)
Protestant	41.5 (0.10)	47.7 (0.16)
Eastern Orthodox	41.3 (0.22)	46.7 (0.40)
Others	41.4 (0.30)	46.9 (0.46)
Subjective Household Income		
Living comfortably	42.1 (0.08)	47.3 (0.11)
Coping	41.8 (0.06)	47.6 (0.10)
Difficult	41.5 (0.10)	46.9 (0.18)
Very Difficult	41.6 (0.18)	46.6 (0.30)

Note: Based on 21 countries; values were weighted based on survey sampling weights provided by the 2006 European Social Survey to better represent the population parameter.

**Table 3:** Social perceptions of maternal and paternal deadline age for childbearing by countries.

Country	Maternal Childbearing Deadline Age	Paternal Childbearing Deadline Age
Austria	43.6	51.1
Belgium	40.9	45.4
Bulgaria	41.1	45.2
Cyprus	42.7	48.4
Denmark	40.5	45.1
Finland	42.7	50.4
France	42.2	47.7
Germany	41.5	47.1
Hungary	39.2	45.8
Ireland	42.3	47.0
Netherlands	40.8	45.8
Norway	41.4	47.0
Poland	40.6	46.6
Portugal	42.7	48.3
Russia	40.9	47.6
Slovenia	42.5	48.9
Spain	42.9	46.0
Sweden	42.5	47.8
Switzerland	41.7	47.1
UK	42.6	48.3
Ukraine	41.8	45.9
Average	41.8	47.3

Source: Data were generated by the author’s own calculation from the 2006 European Social Survey.

**Table 1:** Number of IVF clinics, ART cycles, and ART births by countries.

Country	Num.IVF Clinic	Female Pop. (1000) Per IVF Clinic	Male Pop. (1000) Per IVF Clinic	ART Cycles	Pop. (Million)	ART Cycles Per Pop.	ART Infants	National Births	% of ART Infants
Austria	25	170	160	5177	8.3	624	1041	78,227	1.3
Belgium	18	298	286	22,730	10.5	2165	4019	121,382	3.3
Denmark	22	125	122	12,618	5.4	2337	2674	65,647	4.1
Finland	18	149	143	9116	5.3	1720	1908	59,063	3.2
France	102	318	304	65,749	61.2	1074	13,480	829,000	1.6
Germany	122	345	330	54,695	82.4	664	10,427	675,144	1.5
Netherlands	13	634	623	17,770	16.4	1084	4448	185,913	2.4
Norway	11	213	209	7134	4.7	1518	1660	58,746	2.8
Slovenia	3	342	325	2807	2.0	1404	672	18,649	3.6
Sweden	14	327	320	14,931	9.1	1631	3417	104,495	3.3
Switzerland	24	159	152	7109	7.5	948	1241	73,771	1.7
UK	70	443	422	43,953	60.5	726	12,698	726,000	1.7
Cyprus	7	74	74	1432	1.0	1432			
Bulgaria	15	260	248						
Hungary	10	529	479						
Ireland	7	306	297						
Poland	32	615	577						
Portugal	21	259	242						
Russia	55	1389	1214						
Spain	182	124	118						
Ukraine	16	1573	1366						

Source: Data generated from the 2006 Assisted reproductive technology in Europe report.

**Table 4:** Social perceptions of maternal and paternal deadline age for childbearing by countries.

	Num. of Population Per IVF Clinic		ART Cycles/Population		% of ART Infants	
	Maternal Deadline Age	Paternal Deadline Age	Maternal Deadline Age	Paternal Deadline Age	Maternal Deadline Age	Paternal Deadline Age
	b (SE)	b (SE)	b (SE)	b (SE)	b (SE)	b (SE)
Independent Variable	−3.16 × 10^−3^ (1.46 × 10^−3^) *	3.17 × 10^−3^ (2.64 × 10^−3^)	−1.02 × 10^−3^ (4.48 × 10^−4^) *	−1.43 × 10^−3^ (8.76 × 10^−5^)	−0.48 (0.28)	−0.76 (0.54)
Age-group (ref: 15–24)						
25–34	0.01 (0.18)	0.98 (0.28) ***	−0.23 (0.21)	1.13 (0.32) ***	−0.23 (0.21)	1.11 (0.32) **
35–44	0.43 (0.19) *	2.68 (0.30) ***	0.28 (0.22)	2.87 (0.34) ***	0.27 (0.21)	2.86 (0.34) ***
45–54	−0.01 (0.19)	2.77 (0.30) ***	−0.21 (0.22)	3.17 (0.35) ***	−0.20 (0.22)	3.16 (0.35) ***
55–64	−0.47 (0.20) *	2.98 (0.31) ***	−0.68 (0.23) **	3.21 (0.36) ***	−0.68 (0.23) **	3.19 (0.36) ***
65+	−0.59 (0.19) **	2.92 (0.30) ***	−1.02 (0.23) ***	3.20 (0.35) ***	−1.02 (0.22) ***	3.19 (0.36) ***
Female	−0.14 (0.09)	0.62 (0.14) ***	−0.06 (0.10)	0.66 (0.16) ***	−0.05 (0.10)	0.68 (0.16) ***
Married/Civil partnership	0.06 (0.01) ***	0.14 (0.02) ***	0.05 (0.12) **	0.13 (0.19) ***	0.05 (0.12) **	0.13 (0.03) ***
City residence	0.03 (0.09)	0.13 (0.14)	0.09 (0.10)	0.32 (0.17)	0.09 (0.10)	0.31 (0.17)
Religion (ref: no religion)						
Catholic	0.01 (0.12)	0.34 (0.18)	−0.12 (0.14)	0.20 (0.22)	−0.14 (0.14)	0.20 (0.22)
Protestant	−0.05 (0.14)	0.57 (0.21) **	−4.54 × 10^−4^ (0.14)	0.65 (0.22) **	−0.01 (0.14)	0.65 (0.22) **
Orthodox	−0.41 (0.72)	−0.13 (1.19)	−0.24 (0.77)	−0.01 (1.25)	−0.25 (0.77)	−0.90 (1.25)
Others	−0.35 (0.25)	−0.01 (0.42)	−0.33 (0.27)	0.21 (0.46)	−0.34 (0.27)	0.21 (0.46)
Education (ref: < Lower secondary)						
Lower secondary	0.16 (0.16)	−0.15 (0.26)	0.39 (0.21)	−0.09 (0.32)	0.38 (0.21)	−0.10 (0.32)
Upper secondary	0.04 (0.16)	0.54 (0.25) *	0.33 (0.19)	0.64 (0.31) *	0.32 (0.19)	0.63 (0.31) *
Higher than post-secondary	0.69 (0.17) ***	1.73 (0.26) ***	0.96 (0.20) ***	1.88 (0.32) ***	0.96 (0.20) ***	1.87 (0.32) ***
Number of children (ref: none)						
1	−0.20 (0.15)	−0.93 (0.24) ***	−0.16 (0.17)	−0.98 (0.27) ***	−0.16 (0.17)	−0.97 (0.27) ***
2	−0.39 (0.14) **	−1.27 (0.23) ***	−0.41 (0.16) *	−1.25 (0.26) ***	−0.41 (0.16) *	−1.23 (0.26) ***
3 or more	−0.26 (0.15)	−0.95 (0.25) ***	−0.22 (0.17)	−0.97 (0.27) **	−0.21 (0.17)	−0.95 (0.28) **
Health (ref: Good or very good)						
Fair	−0.42 (0.11) ***	−0.27 (0.17)	−0.31 (0.12) *	−0.32 (0.20)	−0.32 (0.12) *	−0.31 (0.20)
Bad or Very Bad	−0.46 (0.17) **	−0.72 (0.28) *	−0.86 (0.22) ***	−1.07 (0.35) **	−0.85 (0.22) ***	−1.06 (0.35) **
Subjective income (ref: living comfortably)						
Coping	0.02 (0.10)	0.05 (0.16)	0.02 (0.11)	−0.01 (0.17)	0.03 (0.11)	−0.01 (0.17)
Difficult	0.19 (0.15)	0.07 (0.23)	0.24 (0.18)	0.16 (0.28)	0.24 (0.18)	0.16 (0.28)
Very Difficult	0.44 (0.24)	0.37 (0.36)	0.57 (0.31)	−0.221 (0.48)	0.58 (0.31)	−0.22 (0.49)
Female labor force participation (mean-centered)	0.08 (0.04)	0.10 (0.08)	0.05 (0.06)	0.02 (0.11)	0.07 (0.06)	0.08 (0.12)
Family benefit expenditure (mean-centered)	−0.35 (0.30)	0.20 (0.51)	0.44 (0.38)	0.54 (0.74)	0.24 (0.38)	0.487 (0.75)
GDP per capita (mean-centered)	−1.89 × 10^−5^ (1.52 × 10^−5^)	−3.74 × 10^−5^ (2.64 × 10^−5^)	−1.79 × 10^−5^ (1.78 × 10^−5^)	−4.27 × 10^−5^ (3.44 × 10^−5^)	−3.04 × 10^−5^ (1.81 × 10^−5^)	−5.53 × 10^−5^ (3.57 × 10^−5^)
Constant	42.73 (0.56) ***	45.23 (0.96) ***	42.99 (0.68)	46.38 (1.28) ***	43.07 (0.77) ***	46.31 (1.50) ***
Random Effects						
*sd(_cons)*	0.84 (0.15)	1.47 (0.26)	0.77 (0.18)	1.52 (0.33)	0.78 (0.17)	1.56 (0.33)
*sd(Residual)*	4.88 (0.03)	7.40 (0.05)	4.73 (0.03)	7.34 (0.05)	4.73 (0.03)	7.34 (0.05)

* *p* < 0.05, ** *p* < 0.01, *** *p* < 0.001.

## Data Availability

Publicly available datasets were analyzed in this study. This data can be found here: https://www.europeansocialsurvey.org/data/download.html?r=3 (accessed on 15 February 2021) (ESS) and; https://doi.org/10.1093/humrep/deq124 (accessed on 15 February 2021) (In the “Supplementary Data” section; ESHRE).

## Appendix A

**Country**	**ESS Data** **(25 Countries)**	**Number of IVF** **Clinics Data** **(21 Countries)**	**ART Cycles** **Data** **(13 Countries)**	**ART Infants** **Data** **(12 Countries)**
Austria	√	√	√	√
Belgium	√	√	√	√
Denmark	√	√	√	√
Finland	√	√	√	√
France	√	√	√	√
Germany	√	√	√	√
Netherlands	√	√	√	√
Norway	√	√	√	√
Slovenia	√	√	√	√
Sweden	√	√	√	√
Switzerland	√	√	√	√
UK	√	√	√	√
Cyprus	√	√	√	
Bulgaria	√	√		
Hungary	√	√		
Ireland	√	√		
Poland	√	√		
Portugal	√	√		
Russia	√	√		
Spain	√	√		
Ukraine	√	√		
Estonia	√			
Latvia	√			
Romania	√			
Slovakia	√			

## Appendix B

**Country**	**Abbreviation**
Austria	AT
Belgium	BE
Bulgaria	BG
Cyprus	CY
Denmark	DK
Finland	FI
France	FR
Germany	DE
Hungary	HU
Ireland	IE
Netherlands	NL
Norway	NO
Poland	PL
Portugal	PT
Russia	RU
Slovenia	SI
Spain	ES
Sweden	SE
Switzerland	CH
UK	GB
Ukraine	UA
